# An Integrated Pan-Cancer Analysis of ADAMTS12 and Its Potential Implications in Pancreatic Adenocarcinoma

**DOI:** 10.3389/fonc.2022.849717

**Published:** 2022-02-23

**Authors:** Caiyun Song, Jionghuang Chen, Chaolei Zhang, Dapeng Dong

**Affiliations:** ^1^Department of Gastroenterology, Wenzhou People’s Hospital, Wenzhou, China; ^2^Zhejiang Engineering Research Center of Cognitive Healthcare, School of Medicine, Zhejiang University, Hangzhou, China; ^3^Department of Respiratory and Critical Care Medicine, The First Affiliated Hospital of Wenzhou Medical University, Wenzhou, China

**Keywords:** pan-cancer analysis, pancreatic adenocarcinoma, prognosis, biomarker, tumor microenvironment

## Abstract

**Background:**

A Disintegrin and Metallopeptidase with Thrombospondin Type 1 Motif 12 (ADAMTS12), a member of the ADAMTS family of multidomain extracellular protease enzymes, is involved in the progression of many tumors. However, a pan-cancer analysis of this gene has not yet been performed. Its role in pancreatic adenocarcinoma (PAAD) also remains unclear.

**Methods:**

The Cancer Genome Atlas (TCGA) and Genotype-Tissue Expression data (GTEx) databases were used to analyze ADAMTS12 expression in pan-cancer. We assessed the expression, clinical characteristics, prognostic significance, copy number alteration, methylation, and mutation of ADAMTS12 and its correlation with the tumor immune microenvironment. qRT-PCR and immunohistochemistry assays were also performed to validate the expression of ADAMTS12 in PAAD.

**Results:**

Through bioinformatics analysis and preliminary experimental verification, ADAMTS12 was found to be substantially overexpressed in PAAD. High expression level of ADAMTS12 was correlated with worse survival rates in patients with PAAD and high infiltration levels of tumor-associated macrophages, cancer-associated fibroblasts, immune checkpoint proteins, and immunosuppressive genes.

**Conclusion:**

Our findings suggest ADAMTS12 as a potential prognostic biomarker in PAAD. Elevated ADAMTS12 expression may also indicate an immunosuppressive microenvironment.

## Introduction

Pancreatic adenocarcinoma (PAAD) is the most common primary malignancy of the pancreas, with a 5-year survival rate of less than 10% (1). As a major public health challenge, it is of the utmost urgency to explore the potential mechanisms and identify the crucial biomarkers of PAAD. This will facilitate its diagnosis, prognosis, and treatment.

A significant body of evidence suggests that the tumor microenvironment (TME) plays an important role in the progression of PAAD, where it weakens the response of patients with PAAD to treatment (2, 3). Immune cells and stromal cells in the TME, such as tumor-associated macrophages (TAMs) and cancer-associated fibroblasts (CAFs), are generally remodeled by tumor cells to promote tumor progression (4, 5). For example, Ye H et al. reported that TAMs promote the Warburg effect of pancreatic ductal adenocarcinoma by activating the CCL18/NF-kB/VCAM-1 pathway (6). Thus, identifying TME-relative biomarker genes is crucial to elucidating the underlying mechanisms of tumorigenesis and Development.

A Disintegrin and Metallopeptidase with Thrombospondin Type 1 Motif 12 (ADAMTS12), a member of the ADAMTS family of proteins, is involved in the progression of many tumors (4, 7). The elevated expression of ADAMTS12 in colorectal cancer has been found to promote tumor progression through the activation of the Wnt/β-catenin pathway (8). While ADAMTS12 has also been reported to affect the inflammatory response of pancreatitis (9), and also act as a tumor suppressor gene in lung adenocarcinoma (10). However, its role in pan-cancer and especially in PAAD remains unclear.

In this study, the role of ADAMTS12 in pan-cancer was comprehensively analyzed using data from the TCGA database, which includes data on expression, clinical features, prognostic significance, copy number alteration (CNA), DNA methylation level, and genomic mutation. The association between ADAMTS12 expression, the tumor immune microenvironment (TIME), was also assessed. Our findings revealed that high ADAMTS12 expression predicted immunosuppressive TME in PAAD, leading to lower survival rates among patients. Our study implicates the targeting of ADAMTS12 as a potential method of anti-cancer therapy.

## Materials and Methods

### Data Collection

RNAseq data and clinical information from the TCGA, GTEx, and Cancer Cell Line Encyclopedia (CCLE) projects were obtained through the UCSC Xena database (https://xenabrowser.net/datapages/). The mutant information, CNA, and methylation level of ADAMTS12 in pan-cancer were obtained from the cBioportal database (https://www.cbioportal.org/).

### Cell Lines

The human pancreatic cancer cell lines BxPC-3 and CFPAC and the normal pancreatic ductal epithelial cell line HPNE were obtained from the Chinese Academy of Sciences (Shanghai, China). BxPC-3 cells were cultured in RPMI 1640 medium (Hyclone) containing 10% fetal bovine serum (FBS, Gibco, New York, USA). The CFPAC and HPNE cells were cultured in DMEM supplemented with 10% FBS.

### qRT-PCR of ADAMTS12

Total RNA was isolated and purified from PAAD cells and normal pancreatic ductal epithelial cells using the miRNeasy Mini Kit (Qiagen, Maryland, USA), following the manufacturer’s instructions. Quantitative PCR (qPCR) analysis of samples was performed using PrimeScript RT Reagent Kit (Takara, Otsu, Japan), following the manufacturer’s instructions. β-actin was used as the control gene. The target genes and primers were designed as follows: ADAMTS12 forward: 5’-CAGAAAGGACATCTTGCTGG-3’ and reverse: 5’-TCCTGGCAGAAGGTGCATTC-3’; β-actin forward 5’-CCAACCGCGAGAAGATGACC-3’ and reverse 5’-GAGTCCATCACGATGCCAGT-3’. The Student’s t test was used for qRT-PCR experiments.

### Immunohistochemistry

Pancreatic cancer tissues and adjacent normal pancreatic tissues were obtained from pancreatic cancer patients. The research protocols were approved by the Research Ethics Committee of Sir Run Run Shaw Hospital, Zhejiang University School of Medicine (ID: 20210728-31). All participants gave written consent authorizing the analysis of their tissue samples and medical information for scientific research.

After dewaxing and rehydration, tissue sections were put in EDTA solution, and antigen recovery was performed at 100°C for 30 min. After natural cooling to room temperature, an endogenous peroxidase blocker was used to block endogenous peroxidase activity for 15 min. After washing twice in PBS, tissue sections were incubated with the primary antibody anti-ADAMTS12 (ab203102, Abcam); anti-CD 163 (ab182422, Abcam); or anti-TGF beta 1 (AF1027, Affinity), at 4°C overnight. The next day, the tissue sections were washed twice in PBS and were then incubated with a secondary antibody at room temperature for 1 h. The immunohistochemical reactions were visualized with 3,3,0-diaminobenzidine (DAB), and hematoxylin was used for nuclear staining in all tissue sections. The protein levels were evaluated based on the percentage of positive cells and staining intensity (0, negative; 1+, weak; 2+, moderate; 3+, strong).

### Prognostic Significance of ADAMTS12

Kaplan-Meier and univariate Cox regression (uniCox) analyses were used to evaluate the role of ADAMTS12 in the survival of patients with pan-cancer using the R packages “survminer” and “survival.” Specifically, Kaplan-Meier analysis was used to analyze Overall Survival (OS), while univariate cox analysis was used to analyze OS, disease-specific survival (DSS), disease-free interval (DFI), and progression-free interval (PFI).

### Gene Set Enrichment Analysis

The association between ADAMTS12 and all protein-coding mRNAs was evaluated in PAAD, and the Pearson’s correlation coefficients were calculated. Genes correlated with ADAMTS12 expression in PAAD (*p* < 0.05) were ranked from positive correlation to negative correlation and subjected to GSEA using the R package “clusterProfiler”.

### TME Analysis

The R package “ESTIMATE” was used to evaluate stromal and immune scores using TCGA pan-cancer data. Three different methods were employed to evaluate the correlation between ADAMTS12 expression level and the infiltration scores of different immune cells. (1) The TIMER2 database was used to explore the association between ADAMTS12 and the infiltration level of TAMs and CAFs. (2) The infiltration data of 26 immune cells from the TCGA pan-cancer data was downloaded from a published paper (11), and correlation analysis was performed. (3) The infiltration data of 24 immune cells from 33 tumor types on the ImmuCellAI database (http://bioinfo.life.hust.edu.cn/ImmuCellAI#!/) was downloaded, and correlation analysis was also conducted.

## Results

### Expression Analysis of ADAMTS12

**Figure 1:**
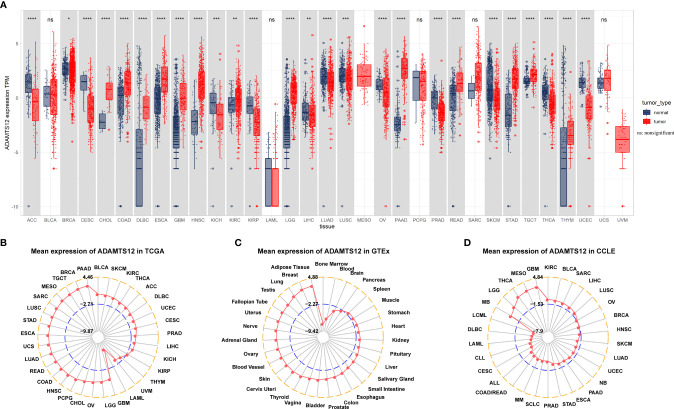
Expression of ADAMTS12. **(A)** Pan-cancer expression of ADAMTS12. **(B)** ADAMTS12 expression in the indicated tumor tissues from the TCGA database. **(C)** ADAMTS12 expression in the indicated human normal tissues from the GTEx database. **(D)** ADAMTS12 expression in the indicated cancer cell lines from the CCLE cohort. *p < 0.05, **p < 0.01, ***p < 0.001, ****p < 0.0001.

The expression of ADAMTS12 was evaluated in 33 tumor types. We observed that ADAMTS12 was overexpressed in 13 of 33 tumor types, which comprise BRCA, CHOL, COAD, DLBC, ESCA, GBM, HNSC, KIRC, LGG, PAAD, READ, STAD, and TGCT (**Figure 1A**). Among tumor tissues from TCGA, we found that ADAMTS12 expression was highest in PAAD and lowest in LAML (**Figure 1B**). For normal human tissues from the GTEx database, ADAMTS12 expression was highest in adipose tissue and lowest in bone marrow tissue (**Figure 1C**). Among cell lines in the CCLE database, ADAMTS12 expression was highest in GBM cell lines and lowest in DLBC cell lines (**Figure 1D**).

**Figure 2:**
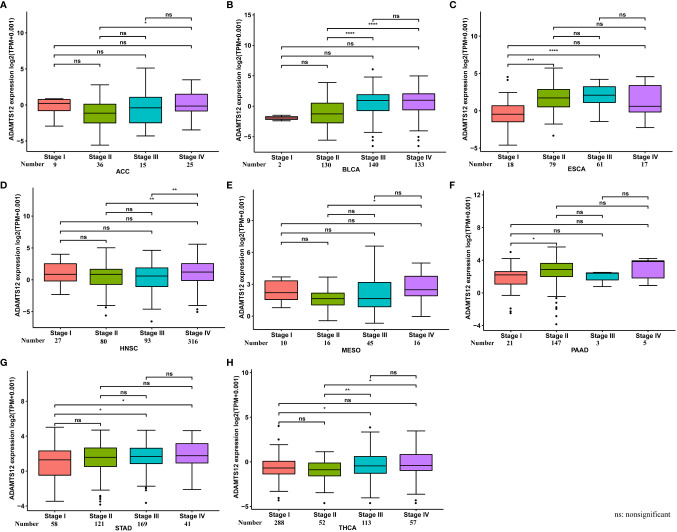
ADAMTS12 expression in different tumor stages. **(A–H)** ADAMTS12 expression in different tumor stages in the indicated tumor types. *p < 0.05, **p < 0.01, ***p < 0.001, ****p < 0.0001.

We further assessed ADAMTS12 expression in various tumor stages. Our results suggest that ADAMTS12 expression was higher in relatively worse tumor stages in ACC, BLCA, ESCA, HNSC, MESO, PAAD, STAD, and THCA (**Figures 2A–H**). For example, the expression of ADAMTS12 in Stage II of PAAD was higher than that in Stage I (**Figure 2F**).

**Figure 3:**
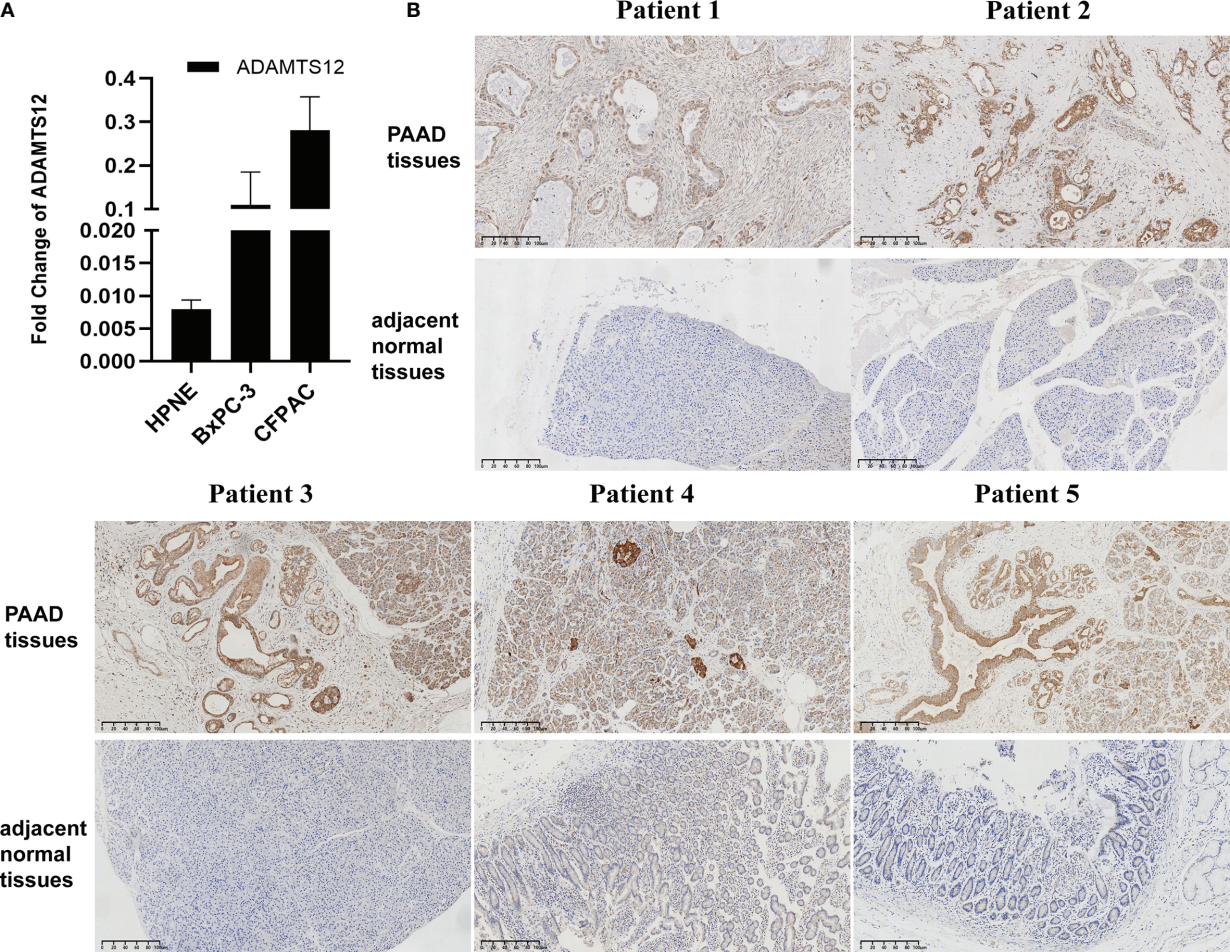
Validation of ADAMTS12 expression. **(A)** qRT-PCR results of ADAMTS12 mRNA expression in the indicated cell lines. **(B)** Immunohistochemistry results of ADAMTS12 protein expression in 5 pairs of PAAD tissues and adjacent normal tissues.

To validate the results from our bioinformatics analyses, we also conducted qRT-PCR and immunohistochemistry assays. Results revealed that the mRNA expression of ADAMTS12 was higher in PAAD cells compared to HPNE cells (**Figure 3A**). Immunohistochemistry also showed a higher level of ADAMTS12 protein in PAAD tissues compared to adjacent normal tissues (**Figure 3B**).

### Gene Alteration of ADAMTS12

**Figure 4:**
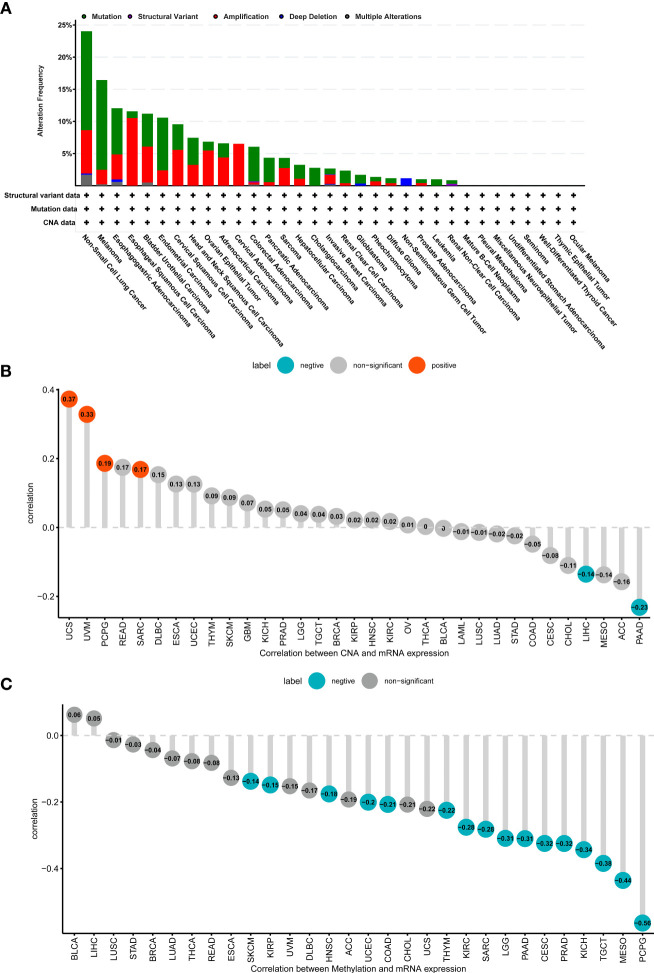
Genomic alteration of ADAMTS12. **(A)** The mutation and CNA status of ADAMTS12 from the TCGA database. **(B)** The association between ADAMTS12 expression and CNA. **(C)** The association between ADAMTS12 expression and DNA methylation.

We evaluated genomic alterations in ADAMTS12, primarily: mutations status, copy number alterations (CNA), and methylation. We found that the genomic alteration frequency of ADAMTS12 was less than 5% in patients with PAAD, in which “Mutation” was the primary type (**Figure 4A**). In addition, the genomic alteration frequency of ADAMTS12 was highest in non-small lung cancer (including LUAD and LUSC), in which “Mutation” was the primary type. Furthermore, we found that ADAMTS12 expression negatively correlates with CNA in PAAD (r = -0.23, *p* < 0.05) (**Figure 4B**). Similarly, the methylation level of the ADAMTS12 promoter was found to be lower in patients with PAAD (r = -0.31), which is likely linked to the elevated expression of the gene (**Figure 4C**).

### Prognostic Significance of ADAMTS12

**Figure 5:**
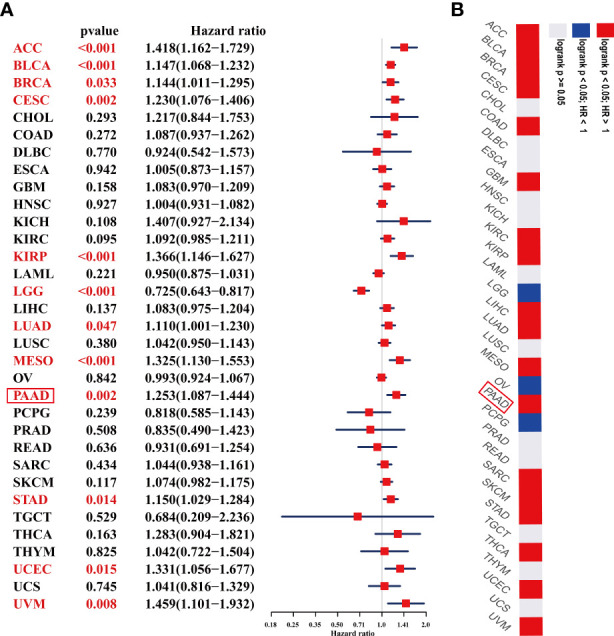
Prognostic value of ADAMTS12 in the OS of patients with PAAD. **(A)** The uniCox OS results of ADAMTS12 in pan-cancer. Significant results (p < 0.05) are in red. **(B)** Kaplan-Meier OS results of ADAMTS12 in pan-cancer. The best cut-off value of ADAMTS12 expression was set. Significant results (*p* < 0.05) are in red.

We further performed UniCox and Kaplan-Meier analyses to evaluate the prognostic significance of ADAMTS12 in pan-cancer. In investigating the prognostic value of ADAMTS12 for OS, the UniCox results indicated that ADAMTS12 was a risk factor in ACC, BLCA, BRCA, CESC, KIRP, LUAD, MESO, PAAD, STAD, UCEC, and UVM. On the other hand, it was found to be a protective factor in LGG (**Figure 5A**). The Kaplan-Meier analysis revealed that high expression of ADAMTS12 predicted worse patient OS in 18 tumor types, comprising ACC, BLCA, BRCA, CESC, COAD, GBM, KIRC, KIRP, LIHC, LUAD, MESO, PAAD, SARC, SKCM, STAD, THCA, UCEC, and UVM (**Figure 5B**, **Supplementary Figure 1**).

We also assessed the prognostic value of ADAMTS12 in disease-specific survival (DSS), disease-free interval (DFI), and progression-free interval (PFI) of tumor patients using UniCox analysis. For DSS analysis, high ADAMTS12 expression predicted shorter DSS times in patients with ACC, BLCA, BRCA, CESC, KICH, KIRP, MESO, PAAD, UCEC, and UVM; longer DSS times were predicted only in LGG (**Table 1**). For DFI, high ADAMTS12 expression predicted shorter DFI times in patients with CESC, KIRP, and PAAD (**Table 2**). Lastly, for PFI, a high ADAMTS12 expression predicted worse PFI status in patients with ACC, BLCA, BRCA, CESC, KICH, KIRP, LUAD, PAAD, and UVM (**Table 3**). These results suggest ADAMTS12 as a potential prognostic biomarker, especially in PAAD (**Supplementary Figure 2**).

**Table 1:** The uniCox results of ADAMTS12 in pan-cancer for DSS.

ID	HR	HR.95L	HR.95H	*p* value
**ACC**	1.459798	1.188414	1.793154	**0.000312**
**BLCA**	1.165799	1.066779	1.274011	**0.000706**
**BRCA**	1.234822	1.042238	1.462992	**0.014762**
**CESC**	1.231566	1.058076	1.433503	**0.007174**
CHOL	1.294942	0.866727	1.934721	0.207043
COAD	1.168453	0.943568	1.446935	0.153469
DLBC	0.814483	0.392411	1.690529	0.581799
ESCA	1.145252	0.960761	1.36517	0.130201
GBM	1.112619	0.986281	1.25514	0.082682
HNSC	1.028969	0.933585	1.134099	0.565044
**KICH**	1.617389	1.011643	2.585838	**0.04461**
**KIRC**	1.130572	0.990459	1.290507	**0.069072**
**KIRP**	1.607481	1.27969	2.019236	**4.51E-05**
LGG	0.735852	0.647532	0.836219	2.58E-06
LIHC	1.060785	0.924388	1.217308	0.400727
LUAD	1.088367	0.956125	1.2389	0.200142
LUSC	1.036973	0.895356	1.200989	0.627965
**MESO**	1.523388	1.201683	1.931215	**0.000505**
OV	0.995187	0.920307	1.076159	0.903776
**PAAD**	1.297255	1.102756	1.52606	**0.001689**
PCPG	0.769106	0.525315	1.126035	0.177115
PRAD	0.5855	0.288238	1.189328	0.138758
READ	0.980187	0.52003	1.84752	0.950658
SARC	1.032547	0.917499	1.162021	0.595148
SKCM	1.094175	0.992939	1.205733	0.06923
STAD	1.083767	0.942047	1.246806	0.260578
TGCT	0.560818	0.174169	1.805814	0.332356
THCA	1.472284	0.884925	2.449494	0.136414
THYM	1.296107	0.802079	2.094424	0.289488
**UCEC**	1.507199	1.144566	1.984724	**0.003483**
UCS	1.068549	0.823205	1.387012	0.618364
**UVM**	1.573389	1.155226	2.142916	**0.004035**

The bold and red font values mean P < 0.05.

**Table 2:** The uniCox results of ADAMTS12 in pan-cancer for DFI.

ID	HR	HR.95L	HR.95H	*p* value
ACC	1.218294	0.916257	1.619897	0.174363
BLCA	0.967268	0.830288	1.126847	0.669272
BRCA	1.179884	0.996035	1.397669	0.05562
**CESC**	1.354509	1.09609	1.673854	**0.004963**
CHOL	1.584944	0.899826	2.791707	0.110821
COAD	0.969077	0.710553	1.321662	0.842731
DLBC	0.787576	0.25729	2.410801	0.675691
ESCA	1.222716	0.944384	1.583078	0.12707
HNSC	1.097528	0.866562	1.390054	0.440149
KICH	2.240898	0.912631	5.502362	0.078325
KIRC	0.912709	0.674571	1.234917	0.553778
**KIRP**	1.383557	1.109285	1.725643	**0.003977**
LGG	0.857034	0.62896	1.167813	0.328433
LIHC	0.957525	0.864845	1.060138	0.403367
LUAD	1.111839	0.962858	1.283872	0.148649
LUSC	0.988583	0.83374	1.172185	0.894898
MESO	2.338984	0.856963	6.383995	0.097186
OV	1.042454	0.943582	1.151686	0.413483
**PAAD**	2.037255	1.386654	2.993112	**0.000289**
PCPG	0.980252	0.58421	1.644774	0.939789
PRAD	1.103614	0.789709	1.542294	0.563694
READ	1.095542	0.469755	2.554972	0.832729
SARC	1.043245	0.902983	1.205294	0.565505
STAD	1.0771	0.877449	1.322179	0.47766
TGCT	1.268267	0.831127	1.935327	0.270404
THCA	1.203681	0.909623	1.5928	0.194575
UCEC	0.897084	0.651791	1.234689	0.505158
UCS	0.853749	0.59611	1.22274	0.388282
ACC	1.218294	0.916257	1.619897	0.174363
BLCA	0.967268	0.830288	1.126847	0.669272
BRCA	1.179884	0.996035	1.397669	0.05562
CESC	1.354509	1.09609	1.673854	0.004963

The bold and red font values mean P < 0.05.

**Table 3:** The uniCox results of ADAMTS12 in pan-cancer for PFI.

ID	HR	HR.95L	HR.95H	*p* value
**ACC**	1.220234	1.043892	1.426365	**0.012442**
**BLCA**	1.105397	1.030905	1.185273	**0.004878**
**BRCA**	1.204577	1.058703	1.37055	**0.004712**
**CESC**	1.249388	1.094664	1.42598	**0.000964**
CHOL	1.072959	0.748264	1.538549	0.701762
COAD	1.130087	0.982213	1.300224	0.087425
DLBC	0.717288	0.449221	1.145322	0.164021
ESCA	1.10262	0.966067	1.258475	0.147563
GBM	1.058525	0.945833	1.184643	0.32202
HNSC	1.002766	0.92673	1.085041	0.945257
**KICH**	1.543453	1.10615	2.153639	**0.010664**
KIRC	1.063237	0.955623	1.182969	0.260066
**KIRP**	1.308359	1.123223	1.524011	**0.000555**
**LGG**	0.82964	0.749692	0.918114	**0.000303**
LIHC	0.984933	0.900352	1.07746	0.740344
**LUAD**	1.110961	1.00906	1.223152	**0.032057**
LUSC	1.036008	0.926414	1.158567	0.535184
MESO	1.087609	0.912281	1.296634	0.349088
OV	1.010346	0.946567	1.078423	0.75703
**PAAD**	1.245042	1.088392	1.424237	**0.0014**
PCPG	0.838423	0.67962	1.034334	0.099994
PRAD	1.119095	0.924991	1.353931	0.246978
READ	1.106471	0.808581	1.514107	0.527233
SARC	1.08453	0.987585	1.190992	0.089418
SKCM	1.027768	0.951693	1.109924	0.48514
STAD	1.059564	0.944777	1.188298	0.322682
TGCT	1.211982	0.838466	1.751891	0.306431
THCA	1.00134	0.815975	1.228814	0.989773
THYM	1.210264	0.97071	1.508936	0.089924
UCEC	1.069057	0.87529	1.305719	0.512805
UCS	1.0443	0.843843	1.292375	0.690182
**UVM**	1.772392	1.342663	2.339659	**5.35E-05**

The bold and red font values mean P < 0.05.

### Enrichment Analysis of ADAMTS12

**Figure 6:**
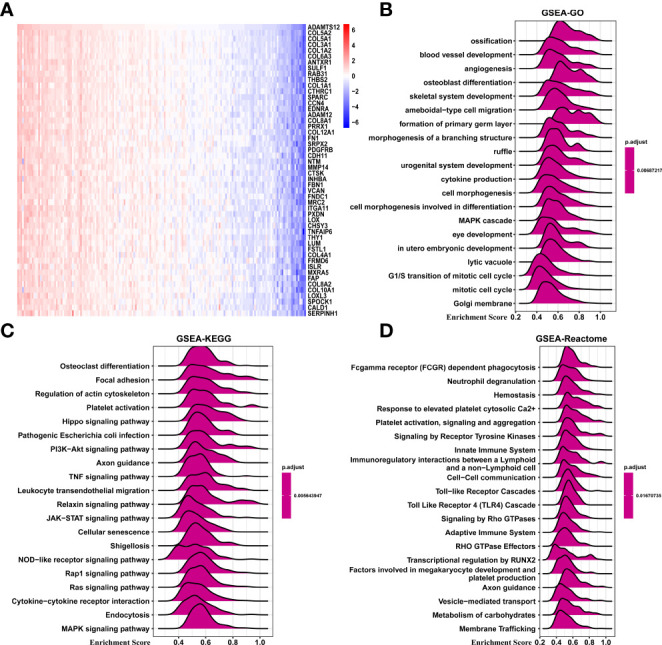
GSEA of ADAMTS12. **(A)** Heatmap representing the expression of the top 50 genes positively correlated with ADAMTS12. **(B)** The top 20 GSEA-GO results were shown in PAAD. **(C)** The top 20 GSEA-KEGG results were shown in PAAD. **(D)** The top 20 GSEA-Reactome results were shown in PAAD.

We performed GSEA to predict the function of ADAMTS12 through correlated genes (**Figure 6A**). According to the correlation analysis, we found that COL5A2, COL5A1, COL3A1, COL1A2, and COL6A3 were most positively correlated with ADAMTS12 expression. Through GSEA-GO analysis, we found ADAMTS12 to mainly be enriched in “ossification,” “blood vessel development,” and “angiogenesis” (**Figure 6B**). From the GSEA-KEGG analysis, ADAMTS12 was observed to be enriched in most tumor-promoting pathways, including the “Hippo signaling pathway,” the “PI3K-Akt signaling pathway,” and the “JAK-STAT signaling pathway” (**Figure 6C**). In the analysis using GSEA-Reactome, ADAMTS12 was observed to be enriched in some immune regulation-related pathways, including “Innate Immune System,” “Immunoregulatory interactions between a Lymphoid and a non−Lymphoid cell,” and “Adaptive Immune System” (**Figure 6D**). We also performed the gene ontology (GO), Kyoto encyclopaedia of genes and genome (KEGG), and Gene set variation analysis (GSVA) in this study (**Supplementary Figure 3**). These results indicate that ADAMTS12 might play a tumor-promoting role and affect the TME.

### TME Analysis

**Figure 7:**
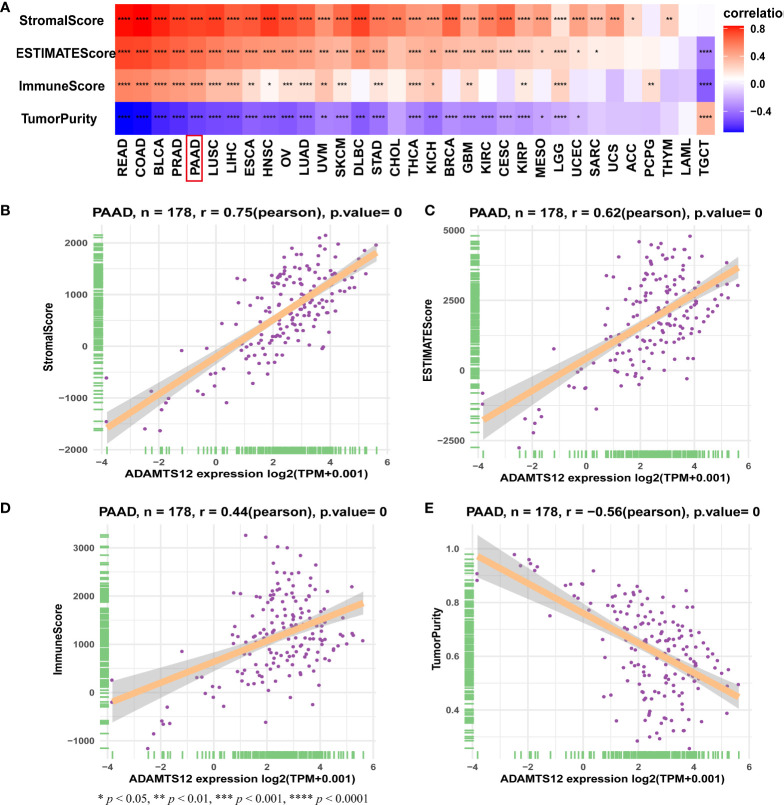
TME scores and ADAMTS12. **(A)** Heatmap representing the correlation between ADAMTS12 expression and TME scores in pan-cancer. **(B–E)** The correlation between ADAMTS12 expression and stromal score **(B)**, ESTIMATE score **(C)**, immune score **(D)**, and tumor purity score **(E)**.

**Figure 8:**
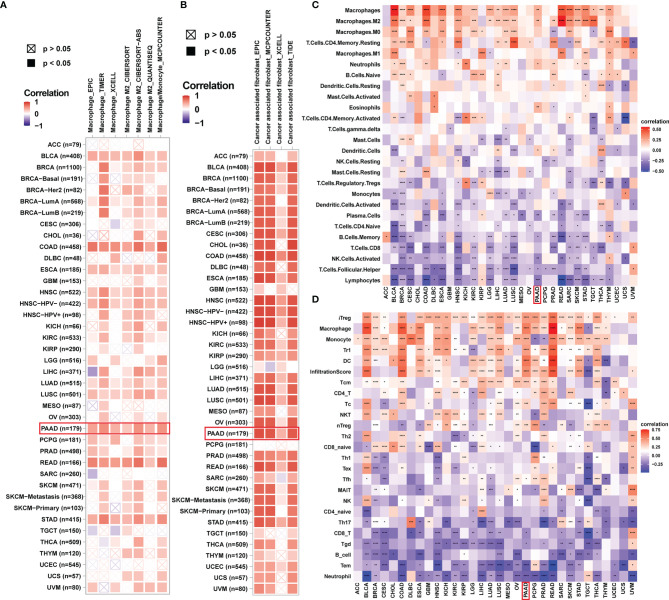
Immune infiltration analysis. **(A, B)** The correlation between ADAMTS12 expression and infiltration levels of TAMs **(A)** and CAFs **(B)** in pan-cancer using the TIMER database. Red represents a positive correlation, while blue represents a negative correlation. The darker the color, the stronger the correlation. **(C)** The correlation between ADAMTS12 expression and the infiltration levels of 26 immune cells from a previous study. **(D)** The correlation between ADAMTS12 expression and the infiltration levels of 24 immune cells from the ImmuCellAI database. *p < 0.05, **p < 0.01, ***p < 0.001, ****p < 0.0001.

**Figure 9:**
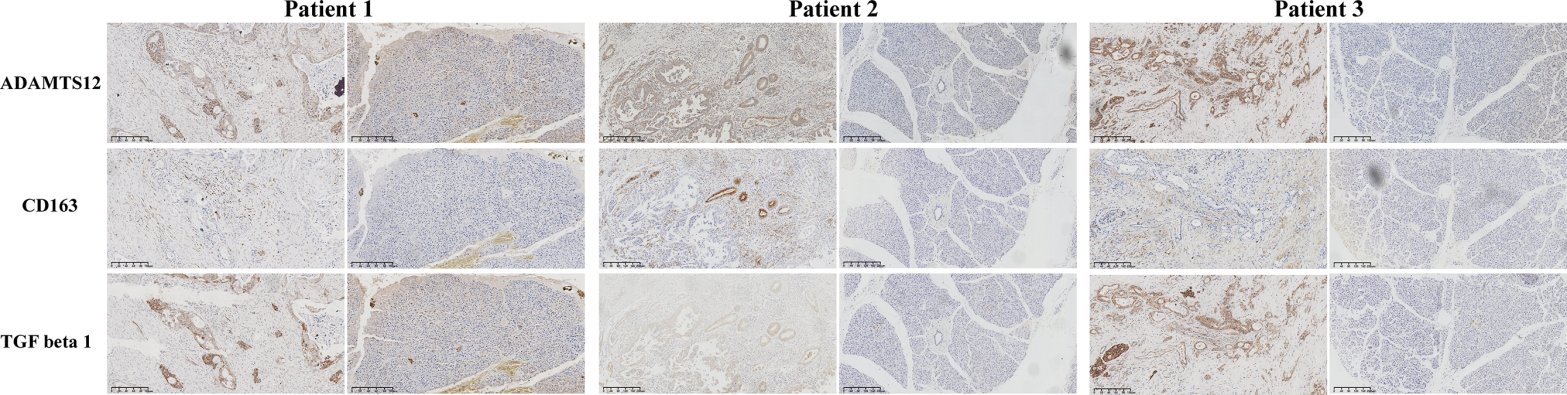
The immunohistochemistry results of ADAMTS12, CD163, and TGF-beta 1 in 3 patients.

In order to understand the role of ADAMTS12 in the TME of PAAD, we analyzed the association between ADAMTS12 expression and stromal and immune scores using the “ESTIMATE” R package. Results indicate that ADAMTS12 is positively correlated with the stromal score, ESTIMATE score, and immune score. On the other hand, the gene is negatively correlated with tumor purity in most tumor types (**Figure 7A**), especially in PAAD (**Figures 7B**–**9**).

To provide evidence for the immune-regulation function of ADAMTS12, we analyzed the correlation between ADAMTS12 expression and immune cell infiltration. Through different methods (including analyzing immune cell infiltration data from a published paper (11), ImmuCellAI database, and TIMER2 database), we found that ADAMTS12 expression correlates positively with the infiltration of immunosuppressive cells (particularly TAMs, CAFs) in most tumor types, including PAAD (**Figures 8A–D**). 3 pairs of PAAD tissues and adjacent normal tissues were evaluated by immunohistochemistry. The immunohistochemistry results of ADAMTS12, CD163, and TGF-beta 1 preliminary confirmed that ADAMTS12 expression correlates positively with the infiltration of TAMs, CAFs (**Figure 9**).

We further revealed that ADAMTS12 expression was positively correlated with immune checkpoint proteins (such as PDCD1, CD274, LAG3, and CTLA4), immunosuppressive genes (**Supplementary Figure 4A**), chemokines (**Supplementary Figure 4B**), and chemokine receptors (**Supplementary Figure 4C**) in most tumor types. We also performed the correlation analysis between ADAMTS12 and the m6A related genes in pan-cancer (**Supplementary Figure 5**). The results indicated that ADAMTS12 are positively correlated with m6A related genes in PAAD. These results imply that Patients with PAAD with high ADAMTS12 expression might be in a relatively immunosuppressive environment.

## Discussion

PAAD is the most common primary malignancy of the pancreas, with a 5-year survival rate of less than 10% (1). Additionally, there is now a significant body of evidence suggesting that the tumor microenvironment (TME) plays a role in the progression of this disease (12, 13). Fabien et al. demonstrated that gene expression changes within the TME of PAAD correlate with tumor progression (4). Thus, it is urgent to elucidate the potential mechanisms of PAAD, identify crucial biomarkers, and explore its relationship with the TME. This is expected to facilitate progression in the diagnosis, prognosis, and treatment of PAAD.

ADAMTS12, a member of the ADAMTS family of proteases, was previously found to affect the progression of many tumors. Moncada-Pazos et al. found that ADAMTS12 is epigenetically silenced in tumor cells and transcriptionally activated in the stroma during progression of colon cancer (7).The expression of ADAMTS12 in colorectal cancer-associated TME prevents cancer development and is a good prognostic indicator of colorectal cancer (14). Hour et al. found that ADAMTS12 displays antiangiogenic properties and protect the host toward tumor progression (15). However, the role of ADAMTS12 in pan-cancer and especially in PAAD remains unclear. In this study, we assessed the expression of ADAMTS12 and found it to be elevated in 13 out of 33 tumor types compared with normal tissues. These tumor types are comprised of BRCA, CHOL, COAD, DLBC, ESCA, GBM, HNSC, KIRC, LGG, PAAD, READ, STAD, and TGCT. Among these, the expression of ADAMTS12 was highest in PAAD. Furthermore, the expression of ADAMTS12 was found to be higher in stage II PAAD than in stage I. The overexpression of ADAMTS12 in PAAD was also confirmed *in vitro* through PAAD cell lines and tissues.

To evaluate the prognostic significance of ADAMTS12, we performed UniCox and Kaplan-Meier analyses in 33 tumor types. UniCox analysis showed that ADAMTS12 is a risk factor for OS, DSS, DFI, and PFI of patients with PAAD. These results provide further evidence for the overexpression of ADAMTS12 in PAAD. Additionally, high ADAMTS12 expression was correlated with lower survival rates in Patients with PAAD. These results also in accordance with other reports such in kidney renal clear cell carcinoma (16) and stomach adenocarcinoma (17, 18). Our results also found that ADAMTS12 is a protective factor in LGG, which was obvious contrary with other results. So far, ADAMTS12 has not been studied in LGG. We believe that this result needs to be further confirmed in LGG in the future.

We also explored potential mechanisms for the effect of ADAMTS12 on prognosis. GSEA of ADAMTS12 revealed that the immune-regulation relevant pathways, such as “Innate Immune System”, “Immunoregulatory interactions between a Lymphoid and a non−Lymphoid cell”, and “Adaptive Immune System”, were enriched in PAAD, indicating an essential role of ADAMTS12 in immune regulation. We further showed that ADAMTS12 is positively correlated with the stromal score, ESTIMATE score, and immune score. On the other hand, we found the gene to be negatively correlated with tumor purity in PAAD.

Different methods were employed to evaluate the relationship between ADAMTS12 and the infiltration levels of immune cells. First, using the TIMER2 database, we showed that ADAMTS12 expression was associated with high infiltration level of TAMs and CAFs. Second, we obtained infiltration data of 26 immune cells from a previous study (11) and performed correlation analysis. The results showed a positive correlation between ADAMTS12 expression and the infiltration of TAMs, especially M2-like TAMs. Third, we downloaded the infiltration data of 24 immune cells from the ImmuCellAI database and found ADAMTS12 expression to be positively correlated with the infiltration levels of TAMs and iTregs. Then we validated the expression of CD 163 (marker of TAMs) and TGF beta 1 (marker of CAFs) in PAAD tissues and adjacent normal tissues. TAMs, especially M2-like TAMs, as a major group of immune cells in TME, were reported playing critical roles in in tumorigenesis and progression process like tumor growth, metastasis, and treatment resistance (5, 19, 20). CAFs are the most essential components of the TME. Numerous previous studies have confirmed the critical role of CAFs in tumorigenesis and development (21–23). The results corroborated the correlation between ADAMTS12 and infiltration of TAMs and CAFs. N6-methyladenosine (m6A) is the most prevalent reversible methylation in mRNA and has critical roles in the tumorigenesis. Our data indicated that ADAMTS12 are positively correlated with m6A related genes in PAAD.

Additionally, we showed that ADAMTS12 expression correlated positively with immune checkpoints and immunosuppressive genes in PAAD, such as CD274, PDCD1, LAG2, CTLA4, and TGFB1. These results reveal that patients with PAAD with high ADAMTS12 expression might be in a relatively immunosuppressed environment.

Our findings revealed ADAMTS12 as a potential prognostic marker in PAAD. High ADAMTS12 expression may contribute to elevated infiltration levels of immunosuppressive cells, such as TAMs, and CAFs.

## Data Availability Statement

The datasets presented in this study can be found in online repositories. The names of the repository/repositories and accession number(s) can be found in the article/**Supplementary Material**.

## Ethics Statement

The studies involving human participants were reviewed and approved by Research Ethics Committee of Sir Run Run Shaw Hospital, Zhejiang University School of Medicine. The patients/participants provided their written informed consent to participate in this study.

## Author Contributions

CS and DD designed the study. CS, JC, and DD performed the analyses and drafted the manuscript. JC and CZ were involved in the validation experiments. All authors read and approved the final manuscript.

## Conflict of Interest

The authors declare that the research was conducted in the absence of any commercial or financial relationships that could be construed as a potential conflict of interest.

The handling editor JC declared a shared parent affiliation with the authors JC, CZ at the time of the review.

## Publisher’s Note

All claims expressed in this article are solely those of the authors and do not necessarily represent those of their affiliated organizations, or those of the publisher, the editors and the reviewers. Any product that may be evaluated in this article, or claim that may be made by its manufacturer, is not guaranteed or endorsed by the publisher.

## Supplementary Material

Supplementary Figure 1 The Kaplan-Meier analysis of ADAMTS12.

Supplementary Figure 2 Prognostic value of ADAMTS12 for the DSS, DFI, and PFI of patients. **(A–C)** The uniCox results of ADAMTS12 in pan-cancer for DSS **(A)**, DFI **(B)**, and PFI **(C)** of patients. Significant results (*p* < 0.05) are in red.

Supplementary Figure 3 GO, KEGG, and GSVA analyses of PAAD and ADAMTS12. **(A)** GO_BP, **(B)** GO_CC, **(C)** GO_MF, **(D)** KEGG, **(E)** Volcano plot of GSVA, **(F)** GSVA.

Supplementary Figure 4 The correlation between the expression of ADAMTS12 and immunosuppressive genes. **(A)** The correlation between ADAMTS12 expression and immunosuppressive genes. **(B–C)** The correlation between ADAMTS12 expression and the expression of chemokines **(B)** and chemokine receptors **(C)**. **p* < 0.05, ***p* < 0.01, ****p* < 0.001, *****p* < 0.0001.

Supplementary Figure 5 The correlation analysis between ADAMTS12 and the m6A related genes in pan-cancer.

## Abbreviations

ACC, Adrenocortical carcinoma; BLCA, Bladder urothelial carcinoma; BRCA, Breast invasive carcinoma; CESC, Cervical squamous cell carcinoma and endocervical adenocarcinoma; CHOL, Cholangiocarcinoma; COAD, Colon adenocarcinoma; DLBC, Lymphoid neoplasm diffuse large B-cell lymphoma; ESCA, Esophageal carcinoma; GBM, Glioblastoma multiforme; HNSC, Head and neck squamous cell carcinoma; KICH, Kidney chromophobe; KIRC, Kidney renal clear cell carcinoma; KIRP, Kidney renal papillary cell carcinoma; LAML, Acute myeloid leukemia; LGG, Lower grade glioma; LIHC, Liver hepatocellular carcinoma; LUAD, Lung adenocarcinoma; LUSC, Lung squamous cell carcinoma; MESO, Mesothelioma; OV, Ovarian serous cystadenocarcinoma; PAAD, Pancreatic adenocarcinoma; PCPG, Pheochromocytoma and Paraganglioma; PRAD, Prostate adenocarcinoma; READ, Rectum adenocarcinoma; SARC, Sarcoma; SKCM, Skin cutaneous melanoma; STAD, Stomach adenocarcinoma; TGCT, Testicular germ cell tumor; THCA, Thyroid carcinoma; THYM, Thymoma; UCEC, Uterine corpus endometrial carcinoma; UCS, Uterine carcinosarcoma; UVM, Uveal melanoma.
