# Supplementary figures and images for: An Integrated Pan-Cancer Analysis of ADAMTS12 and Its Potential Implications in Pancreatic Adenocarcinoma
Source: Front Oncol. 2022 Feb 23;12:849717. doi: 10.3389/fonc.2022.849717 (PMC8904364; doi:10.3389/fonc.2022.849717)

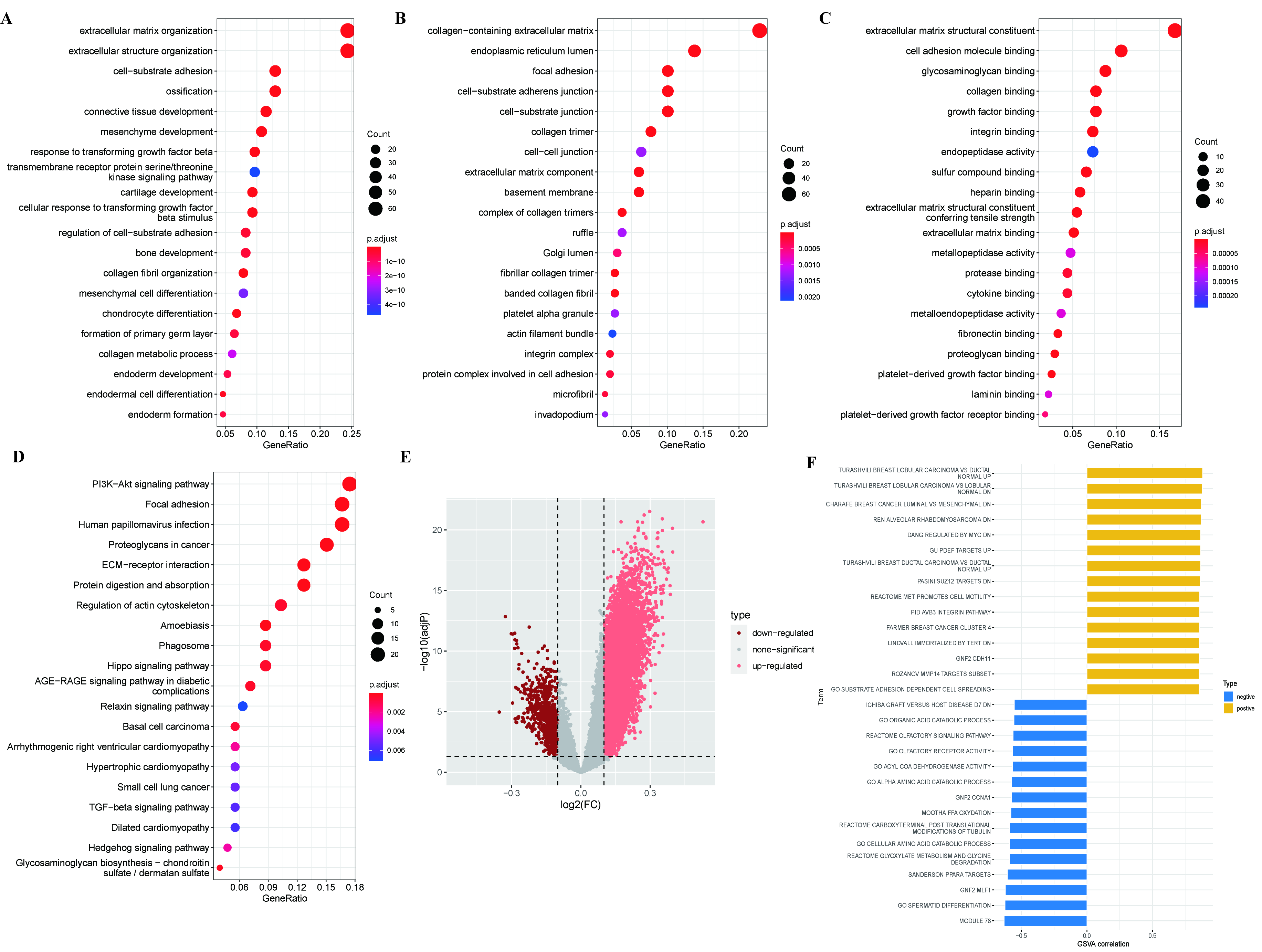

Supplement: Supplementary Figure 3 — GO, KEGG, and GSVA analyses of PAAD and ADAMTS12. (A) GO_BP, (B) GO_CC, (C) GO_MF, (D) KEGG, (E) Volcano plot of GSVA, (F) GSVA. [file Image_3.tif]

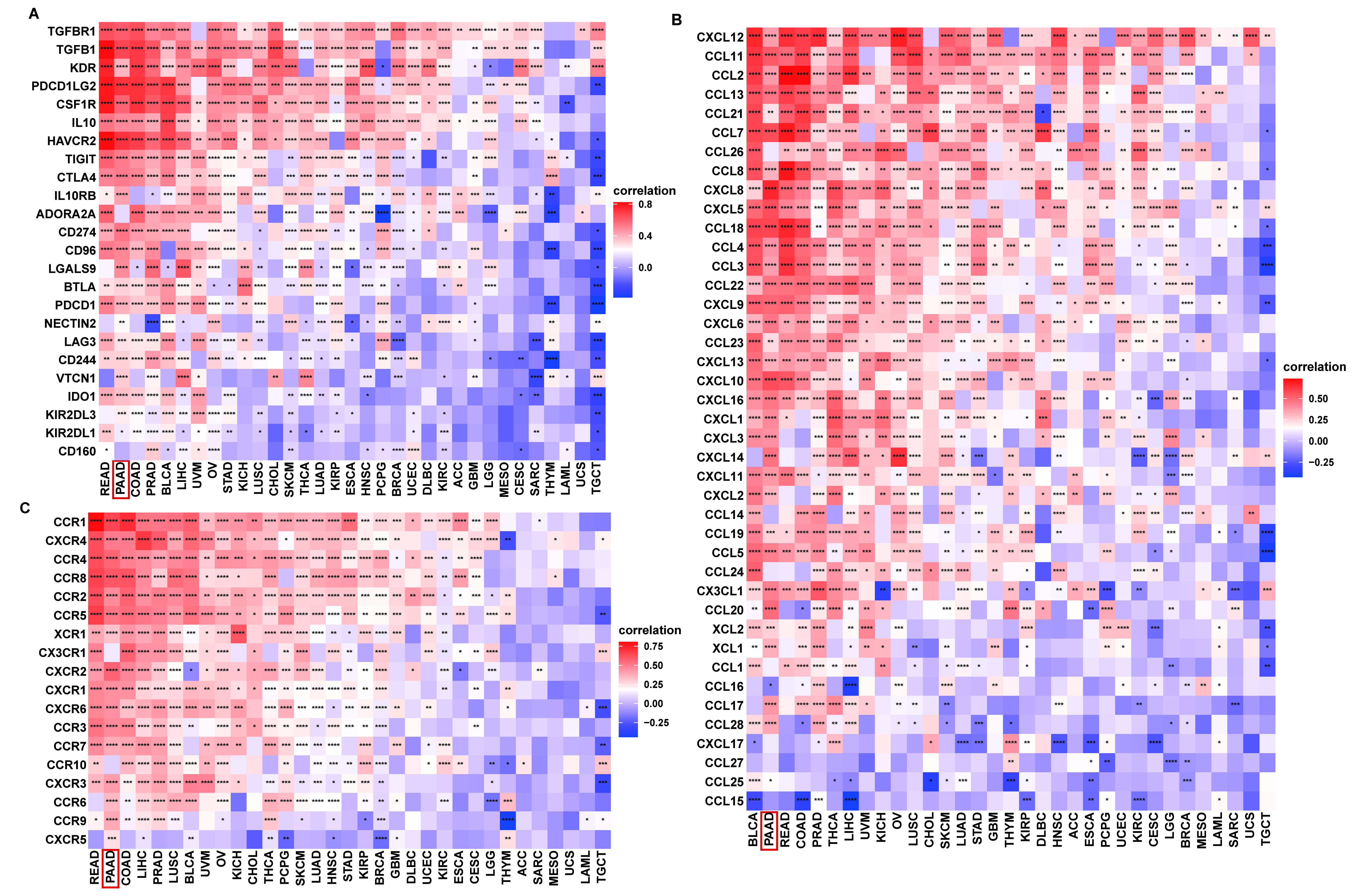

Supplement: Supplementary Figure 4 — The correlation between the expression of ADAMTS12 and immunosuppressive genes. (A) The correlation between ADAMTS12 expression and immunosuppressive genes. (B–C) The correlation between ADAMTS12 expression and the expression of chemokines (B) and chemokine receptors (C). *p < 0.05, **p < 0.01, ***p < 0.001, ****p < 0.0001. [file Image_4.tif]

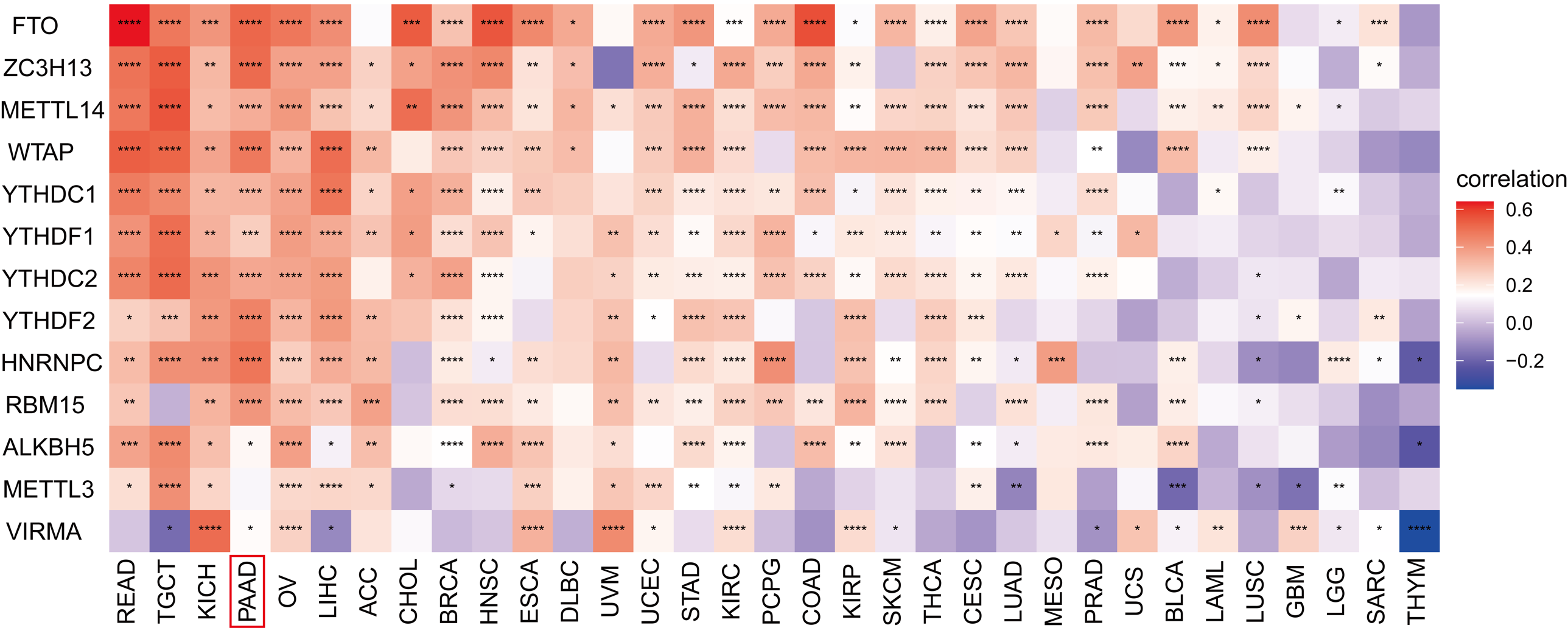

Supplement: Supplementary Figure 5 — The correlation analysis between ADAMTS12 and the m6A related genes in pan-cancer. [file Image_5.tif]
